# Effects of Shading on Starch Pasting Characteristics of Indica Hybrid Rice (*Oryza sativa* L.)

**DOI:** 10.1371/journal.pone.0068220

**Published:** 2013-07-05

**Authors:** Li Wang, Fei Deng, Wan-Jun Ren, Wen-Yu Yang

**Affiliations:** Key Laboratory of Crop Ecophysiology and Farming System in Southwest China, Ministry of Agriculture, Sichuan Agricultural University, Chengdu, People’s Republic of China; China Agricultural University, China

## Abstract

Rice is an important staple crop throughout the world, but environmental stress like low-light conditions can negatively impact crop yield and quality. Using pot experiments and field experiments, we studied the effects of shading on starch pasting viscosity and starch content with six rice varieties for three years, using the Rapid Visco Analyser to measure starch pasting viscosity. Shading at different growth stages and in different rice varieties all affected the starch pasting characteristics of rice. The effects of shading on starch pasting viscosity at middle and later growth stages were greater than those at earlier stages. Shading enhanced breakdown but reduced hold viscosity and setback at tillering-elongation stage. Most pasting parameters changed significantly with shading after elongation stage. Furthermore, the responses of different varieties to shading differed markedly. The change scope of starch pasting viscosity in Dexiang 4103 was rather small after heading, while that in IIyou 498 and Gangyou 906 was small before heading. We observed clear tendencies in peak viscosity, breakdown, and pasting temperature of the five rice varieties with shading in 2010 and 2011. Correlation analysis indicated that the rice amylose content was negatively correlated with breakdown, but was positively correlated with setback. Based on our results, IIyou 498, Gangyou 906, and Dexiang 4103 had higher shade endurance, making these varieties most suitable for high-quality rice cultivation in low-light regions.

## Introduction

Rice (*Oryza sativa* L.) is the staple food for over half of the global population [1] and for about 60% of the population in China [2]. Furthermore, more than 90% of the world’s rice is produced in Asian countries like China, India, Indonesia, Bangladesh, and Viet Nam [3]. However, crop production is affected by environmental stresses, such as heat, drought, salt, and shading. Rice, as a photophilous crop, often encounters low-light stress during the growth stage, particularly in Sichuan, China; the Sichuan Basin receives fewer than 1200 hours of sunshine annually, and only 3345 MJ m^−2^ y^−1^ of annual solar radiation [4].

Light intensity is one of the most important requirements for plant growth, affecting growth, development, survival, and crop productivity. Because of the difficulty of controlling light intensity [5], researchers have evaluated the effects of shading on morphological characteristics, physiological characteristics, yield, and quality of agricultural crops. Multiple studies [6], [7], [8], [9] have shown that the morphological changes resulting from shading included increases in leaf width, length, and area index, and decreases in leaf thickness due to the reduction of palisade layer number, palisade cells, and spongy parenchyma length. Shading also increased thylakoid number in grana and stroma, but reduced trichome density, plastoglobuli, and stomata number [6]. Under the shading treatment applied, the peduncle internode length and plant height increased [7], [10]. Shading generally reduced tiller number and delayed tiller appearance and growing period [11], [12].

In plant photosynthesis, chlorophyll is the most important photosynthetic pigment, and shading also affected the chlorophyll content of plants. Shading altered light-use efficiency by increasing leaf chlorophyll a, chlorophyll b, and chlorophyll a+b, and decreasing chlorophyll a/b ratios [6], [7], [9]. However, differences among plant species exist; for some turfgrass species, chlorophyll content increased in *Lolium perenne* L., decreased in *Poa pratensis* L., but remained unchanged in red fescue (*Festuca rubra* L.) [8]. Furthermore, light intensity changed the rate of non-photochemical quenching, electron transport rate between PSII and PS?, and quantum yield of PSII (Φ PSII) [13].

Shading applied during developmental stages could reduce the plant dry matter accumulation and disturb the redistribution of photosynthetic products from vegetative organs into grains. Ultimately, this could affect total grain yield by reducing panicles, spikelets, filled grains, and grain weight [7], [11], [14]. However, shade before booting stage of rice mainly decreased tiller number and effective panicle number, and little reduction in rice yield was observed [15], [16]. When shade occurred after booting stage, the filled grain percentage and 1000-grain weight decreased, which decreased overall rice yield [16], [17].

To be successful staple crops, crops need to be resistant to varying growing conditions, providing consistent yield and quality under a range of environmental conditions. Starch pasting viscosity, which is tested using a Rapid Visco Analyser (RVA), has long been used in estimating the eating, cooking, and processing quality of rice [18], [19], [20]. While many previous studies focused on shading effects on rice morphology, physiology, and yield, the responses of starch quality to shading in indica hybrid rice are unclear. Therefore, we examined the effects of shading on starch content and starch pasting viscosity in rice genotypes. These research results may lay a theoretical foundation for the selection of shade-tolerant varieties of rice and the improvement of cultivation technologies.

## Materials and Methods

### Plant Materials and Experimental Conditions

The experiments were conducted on the farm of Sichuan Agricultural University in 2009–2011, Ya’an (29°58′N and 102°59′E), Sichuan Province, P. R. China, in a humid monsoon climate. The mean annual accumulated temperature is 6030.4°C, with rainfall of about 1798.6 mm and sunshine hours of about 944.0 h (Tables 1, 2). The soil type of pot and field experiments is a heavy loam (Table 3).

**Table 1:** Meteorological data in 2009, 2010, and 2011.

Meteorological factors	2009	2010	2011
Rainfall during the whole growing period (mm)	1489.4	1845.2	1226.9
Accumulated temperature during the whole growing period (°C)	4035.1	3889.0	4021.4
Sunshine hours during the whole growing period (h)	525.4	523.4	672.9
Rainfall during heading-maturing stage (mm)	323.1	952.4	–
Accumulated temperature during heading-maturing stage (°C)	588.9	1263.8	–
Sunshine hours during heading-maturing stage (h)	81.6	189.7	–
Rainfall during 30 d after heading (mm)	–	–	549.4
Accumulated temperature during 30 d after heading (°C)	–	–	795.5
Sunshine hours during 30 d after heading (h)	–	–	181.6

**Table 2:** Sunshine hours (h) during different growth stages of rice varieties (2009).

Varieties	TE	EB	BH	HM
	(shading time)	(shading time)	(shading time)	(shading time)
Gangyou 906	111.5	52.3	45.0	81.6
	(23 May.–29 June.)	(30 June.–21 July.)	(22 July.–08 Aug.)	(14 Aug.–06 Sept.)
IIyou 498	115.8	–	46.1	–
	(23 May.–30 June.)		(23 July.–11 Aug.)	
Gangyou 188	115.8	–	56.6	–
	(23 May.–30 June.)		(24 July.–13 Aug.)	
Gangyou 527	119.3	–	45.0	–
	(23 May.–01 July.)		(22 July.–09 Aug.)	
Chuanxiang 9838	119.3	–	41.8	–
	(23 May.–01 July.)		(23 July–10 Aug.)	

TE, tillering-elongation stage; EB, elongation-booting stage; BH, booting-heading stage; and HM, heading-maturity stage.

**Table 3:** Soil chemical characteristics of experiments in 2009–2011.

Soil chemical indexes	2009	2010	2011
Organic matter (g kg^−1^)	29.60	19.74	29.52
Total N (g kg^−1^)	0.82	2.14	1.38
Total P (g kg^−1^)	0.36	0.24	0.37
Total K (g kg^−1^)	11.44	27.60	27.06
NaOH hydrolysable N (mg kg^−1^)	165.38	161.47	161.02
Olsen-P (mg kg^−1^)	25.34	82.24	58.37
NH_4_OAc extractable K (mg kg^−1^)	74.70	97.61	118.84

The results of preliminary experiment led to the selection of five rice varieties for the pot experiments in 2009: IIyou 498, Gangyou 188, Gangyou 527, Chuanxiang 9838, and Gangyou 906 (Table 4). On May 23, three similar seedlings (at age of 50 days) were transplanted to pots (25 cm in height and 30 cm in diameter). Each pot contained 10 kg of soil previously fertilized with 0.3 g N, 0.3 g P_2_O_5_, and 0.3 g K_2_O. After transplant, N was spilt-applied, 0.18 g pot^−1^ at mid-tillering and 0.12 g pot^−1^ at panicle initiation. K was applied 0.6 g pot^−1^ at panicle initiation.

**Table 4:** Introduction of indica hybrid rice varieties used in the study.

Varieties	Parents	Breeding institutes
IIyou 498	II-32A×Shuhui 498	Rice Research Institute of Sichuan Agricultural University
Gangyou 527	Gang 46A×Shuhui 527	Rice Research Institute of Sichuan Agricultural University
Gangyou 906	Gang 46A×Ronghui 906	Chengdu Academy of Agriculture and Forestry Sciences
Dexiang 4103	Dexiang 074A×Luhui H103	Sichuan Academy of Agricultural Sciences
Gangyou 188	Gang 46A×Lehui 188	Leshan Agriculture and Animal Husbandry Science
		Research Institute
Chuanxiang 9838	Chuanxiang 29A×Fuhui 838	Sichuan Tianyu Seed Co., Ltd, Crop Research Institute of
		Sichuan Academy of Agricultural Sciences

In Experiment 1, one-layer and two-layer white cotton yarn screens, which shaded about 53% and 73% of the full radiation, respectively, covered the top of Gangyou 906 at tillering-elongation stage (TE; from 23 May to 29 June 2009), elongation-booting stage (EB; from 30 June to 21 July 2009), booting-heading stage (BH; from 22 July to 8 August 2009), and heading-maturity stage (HM; from 14 August to 6 September 2009). In Experiment 2, we studied the responses to shading of starch pasting viscosity of II you 498, Gangyou 188, Gangyou 527, and Chuanxiang 9838 from tillering stage (23 May 2009) to elongation stage (from 30 June to 1 July 2009) and from booting stage (from 22 to 24 July 2009) to heading stage (from 9 to 13 August 2009), by covering with one-layer white cotton yarn screen, which shaded about 53% of the full radiation.

On 20 May 2010 and 25 May 2011, fifty-day-old seedlings were transplanted at a spacing of 33.3 cm×20.0 cm, with two seedlings per hill using plot size of 14.00 m^2^; IIyou 498, Gangyou 188, Gangyou 527, Chuanxiang 9838, and Dexiang 4103 were selected (Table 4). Fertilizer was applied at a rate of 180 kg ha^−1^ of N as urea, 90 kg ha^−1^ of P_2_O_5_ as single superphosphate, and 180 kg ha^−1^ of K_2_O as potassium chloride. N was split-applied at multiple growing stages: 75.6 kg ha^−1^ at basal, 32.4 kg ha^−1^ at mid-tillering, 43.2 kg ha^−1^ at panicle initiation, and 28.8 kg ha^−1^ at booting. P was applied at basal, and K application was split equally at basal and panicle initiation. One-layer white cotton yarn screen, which shaded about 53% of the full radiation, covered the top of the rice canopy from heading (5 August 2010) to maturity (26 September 2010), and from heading (8 August 2011) to 30 d after heading (7 September 2011).

The shading screens were more than 2.0 m above the ground to ensure good ventilation and were large enough to fully cover the shaded plants. Plants without covers were set as controls (CK). The pot experiments were conducted using a randomized design, and all field experiments were in randomized block designs, with three replications. In the rice paddy field, we used a high-efficiency irrigation technique of damp irrigation before booting, rational irrigation during booting, and wetting-drying alternation irrigation after heading. Insects, weeds, and diseases were controlled when required. The water level of each pot was maintained at 1–2 cm in depth, and other rice management actions were similar to those used in the paddy field.

### Seed Collection for Physicochemical Properties Analysis

At maturity, the seeds from the field experiments were randomly selected from five hills in the center of each block; seeds from the pot experiments were randomly selected from three pots with nine plants. All seeds were dried and stored at room temperature for about three months until the physicochemical properties became stable. Then the seeds were shelled, milled, ground to rice flour using CT410 (FOSS SCINO Co., Ltd., China), and sifted through a 0.5-mm screen.

### Starch Pasting Viscosity

Starch pasting viscosity of milled rice flour was determined with the Rapid Visco Analyser using the Super-3, running with Thermal Cycle for Windows software (Newport Scientific Pvt., Ltd., Australia), according to American Association of Cereal Chemists Standard Method 61-02.01 [21]. 3.00 g rice flour (12% moisture basis) was weighed into a new test canister, and then 25.0 ml ultrapure water was added to the flour in the canister. The instrument mixed the flour and water by rotating a paddle at 960 rpm for the first 10 s of the test, after which viscosity was sensed using a constant paddle rotation speed of 160 rpm. The test profile for rice used the following time/temperature cycle [21]: (1) set the idle temperature to 50°C; (2) hold at 50°C for 1.0 min; (3) increase the temperature to 95°C in 3.8 min; (4) hold at 95°C for 2.5 min; (5) decrease the temperature to 50°C in 3.8 min; (6) then hold at 50°C for 1.4 min. Heating and cooling were linearly increased or decreased between profile set points. The instrument was allowed at least 30 min to warm up before being used.

Starch pasting viscosities were described by six parameters: peak viscosity (the maximum hold viscosity, PKV), hold viscosity (the minimum hold viscosity, HPV), final viscosity (the viscosity achieved at the end of the test, CPV), breakdown (peak viscosity minus hold viscosity, BDV), setback (final viscosity minus peak viscosity, SBV), and pasting temperature (PaT) [21]. All the viscosity parameters were expressed in rapid visco units (RVU).

### Starch Contents of Rice Flour in 2011

The starch contents of rice flour were determined by dual-wavelength spectrophotometry [22], [23]. The amylose wavelengths of 565 nm and 484 nm and the amylopectin wavelengths of 550 nm and 743 nm were selected as measuring wavelengths. The total starch content was the sum of amylose and amylopectin contents. The results were reported on a dry weight basis.

### Statistical Analyses

All data were analyzed using the two-way analysis of variance (ANOVA) and the Fisher’s protected least significance difference (LSD) test at p = 0.05 and p = 0.01 [24] for comparisons between growth stages, light intensities, and varieties using SPSS 16.0 (SPSS, Chicago, USA). Correlation analysis was carried out using MS Excel 2003 and SPSS 16.0.

## Results and Discussion

### Effect of Shading on Starch Pasting Viscosity of Rice Flour at Different Growth Stages

We quantified the starch pasting parameters, PKV, HPV, CPV, SBV, BDV, and PaT, of rice at different growth stages (Tables 5, 6). The difference of starch pasting viscosity of Gangyou 906 was caused by light intensity and growth stage; the interaction between these factors had significant (p<0.01) effects on all starch pasting parameters in Experiment 1 (Table 5). Growth stage significantly affected PKV, HPV, SBV, and PaT, while the effect of light intensity was significant for all starch pasting parameters except for HPV (p<0.01). At TE, shading reduced PKV and HPV, but increased CPV, SBV, and BDV. Furthermore, there were significant differences observed in HPV, SBV, and BDV between 73%-shade treatment and the control (CK). PKV and BDV with 53%-shade, and PaT with 73%-shade were higher than the values for CK by 6.1%, 23.9%, and 1.4%, respectively. SBV was 13.1% lower than CK under 53%-shade, but it was 12.7% higher than CK under 73%-shade at EB stage (p<0.05). 53%-shade at BH increased BDV by 10.6% (p<0.05), but decreased PKV, HPV, and CPV. At HM, shading substantially affected the starch pasting viscosity of rice flour, and there were significant (p<0.05) differences between the majority of treatments.

**Table 5:** Effects of shading on starch pasting viscosity of rice flour of Gangyou 906 in Experiment 1 (2009).

Stages	Treatments	PKV (RVU)	HPV (RVU)	CPV (RVU)	SBV (RVU)	BDV (RVU)	PaT (°C)
TE	CK	370.07±8.27a	259.44±12.22a	478.40±12.73a	108.33±6.10b	110.63±5.57b	76.43±0.44a
	53%-shade	369.21±1.00a	253.39±8.34ab	484.67±5.14a	115.46±5.13ab	115.82±9.20ab	76.61±0.21a
	73%-shade	361.29±2.88a	240.03±6.40b	480.50±1.48a	119.21±2.70a	121.26±8.61a	76.41±0.34a
EB	CK	370.07±8.27b	259.44±12.22a	478.40±12.73a	108.33±6.10b	110.63±5.57b	76.43±0.44b
	53%-shade	392.47±1.50a	255.44±2.16a	486.60±2.96a	94.13±2.80c	137.03±2.85a	76.44±0.03b
	73%-shade	369.24±7.82b	267.96±7.03a	491.36±13.26a	122.13±7.47a	101.28±6.12b	77.50±0.25a
BH	CK	370.07±8.27a	259.44±12.22a	478.40±12.73a	108.33±6.10b	110.63±5.57b	76.43±0.44a
	53%-shade	361.25±12.40a	238.90±11.25b	477.11±10.36a	115.86±4.32ab	122.35±1.15a	76.43±0.09a
	73%-shade	360.54±6.44a	253.21±5.40ab	477.93±6.28a	117.39±1.02a	107.33±5.90b	75.92±0.26a
HM	CK	370.07±8.27b	259.41±12.22b	478.40±12.73b	108.33±6.10a	110.63±5.57b	76.43±0.44b
	53%-shade	410.88±6.20a	278.56±8.25a	510.58±1.02a	99.71±7.03ab	132.32±3.10a	76.42±0.50b
	73%-shade	338.94±0.73c	278.56±8.25a	437.10±5.53c	98.15±4.83b	94.72±2.95c	77.65±0.02a
*F*-value	G	6.79**	10.76**	1.99	10.56**	0.94	5.57**
	L	41.68**	0.45	11.48**	7.17**	44.59**	6.25**
	G×L	17.58**	4.10**	12.44**	7.51**	9.83**	6.39**

TE, tillering-elongation stage; EB, elongation-booting stage; BH, booting-heading stage; HM, heading-maturity stage; G, growth stage; L, light intensity; PKV, peak viscosity; HPV, hold viscosity; CPV, final viscosity; SBV, setback; BDV, breakdown; PaT, pasting temperature; and RVU, rapid visco units.

Values in columns represent the significant differences between CK and shading treatments, (p<0.05). Means ± standard, *n* = 3.

** significant at 0.01 level.

**Table 6:** Effects of shading on starch pasting viscosity of rice flour in Experiment 2 (2009).

Varieties	Treatments	PKV (RVU)	HPV (RVU)	CPV (RVU)	SBV (RVU)	BDV (RVU)	PaT (°C)
IIyou 498	CK	369.93±0.63a	231.12±6.01a	441.42±2.79a	71.49±2.63a	138.81±5.43a	78.37±0.04a
	Shade at TE	374.46±10.58a	224.20±3.28a	439.26±5.00a	64.81±7.59a	150.26±10.49a	78.25±0.26a
	Shade at BH	374.22±10.73a	228.28±4.18a	444.63±13.50a	70.40±2.92a	145.94±8.67a	77.98±0.03a
Gangyou 188	CK	351.54±10.06a	208.00±5.79a	375.85±2.36a	24.31±12.40b	143.54±15.65b	78.59±0.75b
	Shade at TE	362.67±5.30a	194.61±12.63ab	373.31±8.13a	10.64±12.19c	165.10±12.95a	78.39±0.02b
	Shade at BH	308.14±3.15b	181.91±8.39b	349.03±6.98b	40.89±5.15a	126.24±7.12c	79.77±0.2a
Gangyou 527	CK	373.61±3.33a	209.49±5.76a	407.33±7.12a	33.72±5.30b	164.13±2.57a	78.34±0.04b
	Shade at TE	380.64±2.57a	203.87±10.82a	397.83±6.71a	17.19±4.43c	176.77±8.41a	78.79±0.50ab
	Shade at BH	231.19±3.57b	135.44±0.97b	281.05±6.26b	49.86±2.70a	95.75±4.47b	79.00±0.22a
Chuanxiang 9838	CK	354.33±13.38a	217.64±19.43a	405.24±10.96b	50.90±2.66b	136.70±6.85a	78.13±0.20b
	Shade at TE	350.87±2.39a	219.75±0.54a	425.33±4.02a	74.46±1.82a	131.13±2.84a	78.50±0.35b
	Shade at BH	302.00±3.17b	196.34±9.59b	382.17±5.06c	80.17±2.28a	105.67±6.59b	79.87±0.36a
*F*-value	V	70.06**	43.62**	238.24**	120.09**	13.22**	8.68**
	G	301.80**	41.27**	146.89**	28.82**	61.83**	20.88**
	V×G	77.86**	12.16**	58.01**	9.10**	14.91**	7.61**

TE, tillering-elongation stage; BH, booting-heading stage; V, variety; G, growth stage; PKV, peak viscosity; HPV, hold viscosity; CPV, final viscosity; SBV, setback; BDV, breakdown; PaT, pasting temperature; and RVU, rapid visco units.

Values in columns represent the significant differences between CK and shading treatments, (p<0.05). Means ± standard, *n* = 3.

** significant at 0.01 level.

In Experiment 2, the variety, growth stage, and the interactions of these factors had highly significant (p<0.01) effects on all starch pasting parameters (Table 6). At TE, shading significantly (p<0.05) reduced SBV of Gangyou 188 and Gangyou 527 by 56.2% and 49.0%, respectively. However, shading increased BDV (15.0%) of Gangyou 188, and CPV (5.0%) and SBV (46.3%) of Chuanxiang 9838. The influence of shading at BH was greater than that at TE. For Gangyou 188, Gangyou 527, and Chuanxiang 9838, shading reduced PKV, HPV, CPV, and BDV by 5.7% to 41.7%, but significantly increased SBV and PaT by 0.9% to 68.2% (p<0.05). Shading at a later growth stage (after heading) may have greater influence than at an earlier stage. Therefore, shading treatments at heading-maturity stage in 2010 and 30 d after heading in 2011 were studied to clarify the responses of starch pasting viscosity to shading.

### Response of Starch Pasting Viscosity to Shading in Different Rice Varieties

During plant growth and development, environmental conditions could impact rice quality [25]. At heading-maturity stage, the changes of starch pasting viscosity were controlled by heredity and environment (Table 7). BDV is related to the starch stability to heat and shear stress, and SBV is related to the recovery of the viscosity during cooling of the heat [21], [25], [26]. The rice with lower SBV and higher BDV showed good eating quality [18], [27]. The effect of variety was significant (p<0.01) for all starch pasting parameters, and the effect of light intensity was significant for all parameters except SBV. There were significant (p<0.05) or highly significant (p<0.01) interactions between light intensity and variety on PKV, HPV, and CPV. The results showed significant (p<0.05) decreases in PKV and BDV of IIyou 498 (2.7% and 10.1%, respectively), but increases in PaT by 1.5%. For Gangyou 188 with shading, PKV, HPV, CPV, and BDV significantly (p<0.05) decreased by 14.5% to 19.8%. PKV, HPV, and CPV of Gangyou 527 were lower (p<0.05) than controls by 4.4% to 5.7%, but PaT was higher. PKV, CPV, and BDV reduced 4.8%, 2.7%, and 11.4%, respectively, in Chuanxiang 9838. In Dexiang 4103, only PKV significantly (p<0.05) increased with shading (1.9%).

**Table 7:** Effects of shading on starch pasting viscosity of rice flour at heading-maturity stage (2010).

Varieties	Treatments	PKV (RVU)	HPV (RVU)	CPV (RVU)	SBV (RVU)	BDV (RVU)	PaT (°C)
IIyou 498	CK	225.38±1.17a	129.75±3.18a	261.25±2.00a	35.88±3.71a	95.63±4.89a	76.45±0.64b
	Shading	219.30±1.12b	133.38±1.71a	257.54±3.59a	38.17±4.71a	86.00±0.59b	77.60±0.07a
Gangyou 188	CK	210.42±1.30a	122.42±2.83a	244.42±0.71a	34.00±0.59a	88.00±4.12a	77.38±0.60a
	Shading	173.42±2.59b	98.17±1.41b	202.59±1.30b	29.17±3.89a	75.25±4.01b	78.33±0.11a
Gangyou 527	CK	215.13±0.06a	119.00±3.30a	241.54±1.12a	26.42±1.06a	96.13±3.24a	77.70±0.07b
	Shading	205.59±0.82b	112.25±1.30b	227.71±1.94b	22.13±1.12a	93.34±0.47a	78.83±0.67a
Chuanxiang 9838	CK	232.50±0.94a	127.63±1.71a	260.42±1.89a	27.92±0.94a	104.88±0.77a	76.88±0.11a
	Shading	221.25±1.65b	128.38±4.89a	253.50±6.25b	32.25±7.90a	92.88±6.54b	77.70±0.07a
Dexiang 4103	CK	216.04±1.36b	92.00±1.18a	171.38±1.00a	−44.67±0.35a	124.05±0.18a	71.30±0.07a
	Shading	220.13±4.30a	97.09±0.12a	171.88±1.24a	−48.25±5.54a	123.05±4.42a	71.68±0.67a
*F*-value	L	290.34**	15.14**	170.89**	0.94	31.02**	20.60**
	V	306.89**	143.45**	998.46**	613.49**	104.77**	162.38**
	L×V	94.31**	23.67**	56.13*	2.23	3.11	0.52

L, light intensity; V, variety; PKV, peak viscosity; HPV, hold viscosity; CPV, final viscosity; SBV, setback; BDV, breakdown; PaT, pasting temperature; and RVU, rapid visco units.

Values in columns represent the significant differences between CK and shading treatments, (p<0.05). Means ± standard, *n* = 2.

** significant at 0.01 level;

* ,significant at 0.05 level.

Shading at heading-maturity stage (after heading) could significantly decrease BDV of IIyou 498, Gangyou 188, and Chuanxiang 9838, and the rice viscosity was hard. Compared with other rice varieties, Gangyou 527 and Dexiang 4103 were less affected by shading, as their SBV and BDV had no significant differences among different treatments.

The analysis of variance showed that the effect of variety during 30 d after heading was significant (p<0.01) for starch pasting parameters; light intensity also caused significant differences (Table 8). The interactive effects of light intensity and variety had significant influence on starch pasting parameters (p<0.01), except for HPV. For IIyou 498, shading increased SBV by 85.5%, and it decreased PKV (9.2%), HPV (6.5%), CPV (1.9%), and BDV (12.8%). PKV and BDV of Gangyou 188 with shading were significantly (p<0.05) lower than these of controls by 13.4% and 29.7%, respectively, but the other parameters were higher by 3.2% to 101.8%. In Gangyou 527, shading significantly (p<0.05) decreased PKV, HPV, and BDV by 5.0% to 15.3%, and shading increased SBV and PaT by 30.3% and 1.0%, respectively. PKV, HPV, CPV, and SBV of Chuanxiang 9838 were significantly (p<0.05) lower than CK by 2.3% to 12.0%, but PaT was higher by 1.3%. For Dexiang 4103, shading significantly (p<0.05) decreased PKV (8.1%), HPV (5.5%), CPV (4.1%), and BDV (9.9%), but increased SBV by 18.0%. With shading during 30 d after heading, the rice viscosity of IIyou 498, Gangyou 188, Gangyou 527, and Dexiang 4103 were hard with increasing of SBV and decreasing of BDV, but that of Chuanxiang 9838 was softened.

**Table 8:** Effects of shading on starch pasting viscosity of rice flour during 30 d after heading (2011).

Varieties	Treatments	PKV (RVU)	HPV (RVU)	CPV (RVU)	SBV (RVU)	BDV (RVU)	PaT (°C)
IIyou 498	CK	258.08±2.34a	148.47±2.79a	279.58±3.98a	21.50±2.30b	109.61±1.57a	77.55±0.05a
	Shading	234.42±2.73b	138.89±6.77b	274.31±3.90b	39.89±5.19a	95.53±8.56b	77.57±0.10a
Gangyou 188	CK	223.06±2.96a	136.50±4.33a	281.44±2.04b	58.39±0.92b	86.56±2.28a	78.28±0.03b
	Shading	193.14±2.12b	132.25±4.45a	310.97±1.93a	117.83±3.99a	60.89±6.56b	80.78±0.03a
Gangyou 527	CK	211.75±2.82a	118.75±6.17a	264.67±3.84a	52.92±3.06b	93.00±4.45a	79.95±0.05b
	Shading	191.58±0.29b	112.83±3.56b	260.56±2.36a	68.97±2.56a	78.75±3.77b	80.78±0.03a
Chuanxiang 9838	CK	228.64±1.55a	130.17±7.30a	292.61±7.68a	63.97±6.78a	98.47±6.84a	78.32±0.03b
	Shading	223.31±1.75b	122.28±1.35b	279.58±1.61b	56.28±2.80b	101.03±2.03a	79.37±0.03a
Dexiang 4103	CK	247.03±2.95a	97.78±6.83a	175.14±7.17a	−71.89±4.25b	149.25±3.90a	71.80±0.09a
	Shading	226.92±3.17b	92.42±1.96b	167.94±1.73b	−58.97±1.54a	134.50±1.23b	72.10±0.56a
*F*-value	L	623.92**	46.55**	0.00	271.52**	92.30**	181.55**
	V	449.79**	298.79**	1951.51**	1966.68**	278.84**	1842.82**
	L×V	25.90**	0.97	54.96**	82.61**	10.74**	38.21**

L, light intensity; V, variety; PKV, peak viscosity; HPV, hold viscosity; CPV, final viscosity; SBV, setback; BDV, breakdown; PaT, pasting temperature; and RVU, rapid visco units.

Values in columns represent the significant differences between CK and shading treatments, (p<0.05). Means ± standard, *n* = 3.

** significant at 0.01 level.

The shaded rice plants had higher chlorophyll content and larger leaf area before heading [7], [9], [15] and exhibited higher photosynthetic rates than the controls. These changes were beneficial to the accumulation of carbohydrates after regaining normal light. In general, shading reduced the tiller number [11], [12] and increased the percentage of degenerated spikelets [14], resulting in lower effective panicles and filled grains. Shading had less influence on the ultimate brown rice mass and grain yield [15], [16], [17], due to increases in the supply capacity and storage capacity in rice [7], [9], [11], [12]. However, shading after heading seriously reduced the photosynthetic rate of the functional leaves and the quantity of photosynthetic products transported to grain [11], [12], [13]; these reductions were unfavorable for grain filling [7], [14], [17].

The experimental results in 2009–2011 showed that the effect of shading on starch pasting viscosity after heading (30 d after heading and heading-maturity stage) was stronger than that at booting-heading stage, elongation-booting stage, and tillering-elongation stage. Also, different rice cultivars exhibited different levels of sensitivity to shading treatment [9], [11], [12], [13], [15], and these differences manifested themselves at different growth stages in the different rice varieties. At tillering-elongation stage, shading had more influence on Gangyou 188 with lower SBV and higher BDV, and Chuanxiang 9838 with higher SBV and lower BDV (Tables 5, 6). When shading occurred at booting-heading stage, the rice viscosity of Gangyou 188, Gangyou 527, Chuanxiang 9838, and Gangyou 906 was hard, with higher SBV and lower BDV (Tables 5, 6). After heading, BDV of IIyou 498, Gangyou 188, and Gangyou 527 decreased, and the rice viscosity was hard (Tables 7, 8).

The starch pasting viscosity of rice flour, a pasting curve, is generated in a standard temperature program of “heat-hold-cool-hold” [21] and has been used to assist in selecting rice varieties with desirable cooking and eating quality [18], [19], [20]. Starch pasting viscosity, a quantitative trait, was mainly controlled by heredity, although environment affected it to a lesser extent [28], [29]. And the stabilities for the viscosity parameters differed among different rice varieties [30]. Shading generally resulted in an increase in PaT, such as in IIyou 498 in 2010, Gangyou 188 and Chuanxiang 9838 in 2011, and Gangyou 527 in both years (Tables 7, 8). Lower PKV, CPV, and BDV of IIyou 498 and Gangyou 527 were observed with shading across years. Although some of the six viscosity parameters had different change tendencies between 2010 and 2011, we observed stable tendencies in PKV, BDV, and PaT of the five rice varieties with shading across years. Furthermore, the stability of varieties differed, with Dexiang 4103 showing higher shade endurance and stability, but Gangyou 188 and Chuanxiang 9838 showing poor stability.

### Starch Content of Rice Flour

Starch was composed of two forms, amylose and amylopectin, and the amylose content had an effect in determining the physical and chemical properties of rice [18]. The differences of amylose, amylopectin, and total starch contents were caused by heredity, environment, and the interactions of heredity and environment (Table 9). The variety and interactions of light and variety had significant (p<0.05) or highly significant (p<0.01) effect on amylose, amylopectin, and total starch contents; light significantly affected amylopectin and total starch contents. With shading during 30 d after heading, amylose, amylopectin, and total starch contents of Gangyou 188 and Dexiang 4103 increased significantly (p<0.05) by 5.7% to 67.0%, while amylopectin and total starch contents of Chuanxiang 9838 increased 12.0% and 7.6%. Conversely, amylose content of Gangyou 527 decreased 3.9% (p<0.05). The changes of rice starch contents to shading might be related to starch synthesis enzyme activities, such as ADP-glucose pyrophosphorylase, starch branching enzyme, and starch debranching enzyme [31].

**Table 9:** Effects of shading on starch content of rice flour (2011).

Varieties	Treatments	Amylose (%)	Amylopectin (%)	Total starch (%)
IIyou 498	CK	30.52±0.96a	46.64±1.13a	77.17±2.07a
	Shading	30.09±0.60a	44.36±2.78a	74.46±2.43a
Gangyou 188	CK	31.29±0.25b	43.59±1.21b	74.89±1.44b
	Shading	33.07±0.82a	57.12±1.83a	90.19±1.45a
Gangyou 527	CK	27.36±0.37a	52.23±2.10a	79.58±1.87a
	Shading	26.30±1.04b	54.25±5.65a	80.54±4.77a
Chuanxiang 9838	CK	28.87±0.45a	57.67±2.37b	86.54±2.82b
	Shading	28.56±0.45a	64.59±2.82a	93.13±3.17a
Dexiang 4103	CK	20.96±0.48b	42.04±0.93b	63.00±1.41b
	Shading	22.98±0.60a	70.19±2.38a	93.18±2.66a
*F*-value	L	3.92	100.12**	109.48**
	V	283.27**	29.41**	24.21**
	L×V	9.30*	30.41**	32.43**

L, light intensity; and V, variety.

Values in columns represent the significant differences between CK and shading treatments, (p<0.05). Means ± standard, *n* = 3.

** significant at 0.01 level;

* ,significant at 0.05 level.

Cooked rice with high amylose content was rigid, while rice with low amylose content was relatively soft and sticky [18]. Amylose content and amylopectin content were closely related to the starch pasting profile [18], [25]. In our study (Table 10), HPV, CPV, SBV, and PaT were significantly (p<0.01) positively correlated with amylose content (*r* = 0.899, *r* = 0.928, *r* = 0.846, and *r* = 0.747, respectively). A significant (p<0.01) negative correlation between BDV and amylose content (*r* = −0.817) was observed, since higher BDV and lower SBV and amylose content are indicative of good rice quality [18]. However, a negative correlation existed between some starch pasting parameters and amylopectin content, except for SBV and PaT. PKV, HPV, and BDV were negatively correlated with total starch content. Therefore, shading may not only influence morphology, physiology, and yield of rice [7], [9], [11], [14], but may also influence the eating and cooking quality of rice.

**Table 10:** Correlation coefficients between starch pasting viscosity and starch content of rice (2011).

Items	PKV	HPV	CPV	SBV	BDV	PaT
Amylose	−0.228	0.899**	0.928**	0.846**	−0.817**	0.747**
Amylopectin	−0.362	−0.409	−0.126	0.027	−0.010	0.008
Total starch	−0.443	−0.049	0.238	0.356	−0.328	0.299

PKV, peak viscosity; HPV, hold viscosity; CPV, final viscosity; SBV, setback; BDV, breakdown; and PaT, pasting temperature.

** significant at 0.01 level.

## Conclusions

Heredity, environment, and the interactions of heredity and environment were combined to affect starch pasting viscosities and starch contents of different rice varieties. In our study, shading at earlier growth stages had less effect on starch than did shading at later growth stages. At later growth stages, shading resulted in decreased peak viscosity and breakdown, but increased pasting temperature. Furthermore, different rice varieties responded differently to shading. Gangyou 188, Gangyou 527, and Chuanxiang 9838 exhibited the greatest changes due to shading. IIyou 498 and Gangyou 906 had higher endurance to shading before heading, while Dexiang 4103 could maintain high quality when exposed to shade after heading. These differences in the shade endurance among rice varieties can offer a theoretical foundation for selecting and breeding shade-tolerant rice. Using this approach, rice quality would be enhanced by using reasonable cultivation technologies and selecting varieties with strong shade endurance in the low-light regions.

Light illumination has complex effects on rice grain quality. Shading not only affects the filling rate, carbohydrate accumulation of grain, and dry matter transportation in stem-sheath, but it also affects starch synthase and related enzyme activities. Therefore, the relationship between key enzyme activity of starch and starch pasting characteristics, and the technique of rice breeding and cultivation to improve shade endurance require further investigation.
